# HutZ is required for biofilm formation and contributes to the pathogenicity of *Edwardsiella piscicida*

**DOI:** 10.1186/s13567-019-0693-4

**Published:** 2019-10-02

**Authors:** Yan-Jie Shi, Qing-Jian Fang, Hui-Qin Huang, Chun-Guang Gong, Yong-Hua Hu

**Affiliations:** 1 Ocean College of Hebei Agricultural University, Qinhuangdao, 066000 China; 2 Institute of Tropical Bioscience and Biotechnology, Chinese Academy of Tropical Agricultural Sciences, Haikou, 571101 China; 3 Laboratory for Marine Biology and Biotechnology, Pilot National Laboratory for Marine Science and Technology, Qingdao, China; 4 Hainan Provincial Key Laboratory for Functional Components Research and Utilization of Marine Bio-resources, Haikou, 571101 China

## Abstract

*Edwardsiella piscicida* is a severe fish pathogen. Haem utilization systems play an important role in bacterial adversity adaptation and pathogenicity. In this study, a speculative haem utilization protein, HutZ_Ep_, was characterized in *E. piscicida*. *hutZ*_*Ep*_ is encoded with two other genes, *hutW* and *hutX*, in an operon that is similar to the haem utilization operon *hutWXZ* identified in *V. cholerae*. However, protein activity analysis showed that HutZ_Ep_ is probably not related to hemin utilization. To explore the biological role of HutZ_Ep_, a markerless *hutZ*_*Ep*_ in-frame mutant strain, TX01Δ*hutZ*, was constructed. Deletion of *hutZ*_*Ep*_ did not significantly affect bacterial growth in normal medium, in iron-deficient conditions, or in the presence of haem but significantly retarded bacterial biofilm growth. The expression of known genes related to biofilm growth was not affected by *hutZ*_*Ep*_ deletion, which indicated that HutZ_Ep_ was probably a novel factor promoting biofilm formation in *E. piscicida*. Compared to the wild-type TX01, TX01Δ*hutZ* exhibited markedly compromised tolerance to acid stress and host serum stress. Pathogenicity analysis showed that inactivation of *hutZ*_*Ep*_ significantly impaired the ability of *E. piscicida* to invade and reproduce in host cells and to infect host tissue. In contrast to TX01, TX01Δ*hutZ* was defective in blocking host macrophage activation. The expression of *hutZ*_*Ep*_ was directly regulated by the ferric uptake regulator Fur. This study is the first functional characterization of HutZ in a fish pathogen, and these findings suggested that HutZ_Ep_ is essential for *E. piscicida* biofilm formation and contributes to host infection.

## Introduction

Iron is an essential element for bacteria because it is necessary for a wide variety of physiological processes, including electron transfer, enzyme catalysis, energy transduction, and regulation of gene expression [1, 2]. Iron also plays a key role in host–pathogen interactions in animals and plants, so iron is necessary for bacterial invasion and successful infection [3, 4]. Although iron is the most abundant metallic element on earth, the majority of iron is sequestered in iron- and haem-containing proteins within the host, so iron deficiency is the most common nutritional stress for bacteria [5, 6]. Therefore, bacterial pathogens have developed a variety of strategies that facilitate the uptake and utilization of iron [1, 3]. Since the overwhelming majority of iron in the host is present as haem iron [7], haem is a dominant iron source for most pathogenic bacteria [7, 8]. It is not surprising that many bacterial pathogens have evolved elaborate strategies to acquire haem from host sources, which are important for pathogenesis [7, 9]. One of these strategies is haem uptake systems, and the utilization of haem is a common mechanism employed by pathogens [10].

Haem uptake systems in gram-negative bacteria consist of outer membrane receptors that either directly bind haem and haemoproteins or bind haem-bound secreted haemophores. Haem then transits the periplasm and is brought into the cell via ABC transporters in the inner membrane [9]. There are several types of mechanisms for haem uptake and utilization in gram-negative bacteria. A universal haem uptake system usually involves outer membrane receptors, a TonB-dependent internalization process, a periplasmic binding protein, and an inner membrane-associated ABC transporter, which has been identified in numerous species, including *Escherichia coli*, *Vibrio cholerae*, and *Vibrio anguillarum* [11]. Another mechanism for haem uptake is mediated by a haem-binding outer membrane lipoprotein, as in *Haemophilus influenzae* [12]. The opportunistic pathogen *Pseudomonas aeruginosa* encodes direct haem uptake and haemophore systems at the outer membrane [13], and *Neisseria meningitidis* uses a unique bipartite receptor for haem acquisition from host haemoproteins [14].

The mechanism of haem transfer from outside the cell to the cytoplasm of bacteria has been extensively studied; however, little is known about the fate of haem after it enters the cytoplasm. A haem utilization operon, *hutWXZ*, has been identified in *V. cholerae* [15–17]. A similar operon, *hugWXZ*, was also identified in *Plesiomonas shigelloides* [18]. *hutWXZ* and *hugWXZ* were considered necessary for obtaining iron from haem [17, 18]. In *E. coli*, a haem utilization gene cluster, *chu*, was identified that encodes a series of proteins, including ChuS, ChuA, ChuT, ChuW, ChuX, ChuY, and ChuU [19, 20]. ChuW and ChuX are homologous to HutW and HutX, which constitute the ChuW_HutW and ChuX_HutX superfamilies, respectively. HutW belongs to the S-adenosylmethionine (SAM) radical superfamily and was predicted to serve as an electron carrier for HutZ [17]. ChuW is a radical S-adenosylmethionine methyltransferase that catalyses a radical-mediated mechanism facilitating iron liberation and the production of the tetrapyrrole product termed “anaerobilin”, which can be used as a substrate by ChuY [21]. HutX is a cytoplasmic haem transport protein for HutZ, and haem is transferred from HutX to HutZ via a specific protein–protein interaction [17]. ChuX binds haem with a stoichiometry of 1:1, and ChuX is characterized as a haem-trafficking protein [19]. The third protein of the HutWXZ system in *V. cholerae*, HutZ, is a cytoplasmic haem-binding protein that has been identified as a haem-degrading enzyme [17]. However, ChuY, the counterpart of HutZ, has relatively low homology with HutZ. ChuY has high structural homology with human biliverdin and flavin reductase. It has been reported that ChuY has flavin mononucleotide (FMN) reductase activity, using NAD(P)H as a cofactor, and shows porphyrin ring binding affinity [19, 20]. Moreover, ChuY acts as a reductase in haem homeostasis to maintain the virulence potential of *E. coli* CFT073 [21].

*Edwardsiella piscicida* (formerly included in the *Edwardsiella tarda* species) [22, 23], a family member of Enterobacteriaceae, is a serious fish pathogen and has a broad host range that includes many species of economically important fish, such as Japanese eel, flounder, turbot, red sea bream, tilapia, and channel catfish [24]. Recently, an increasing number of studies on *E. piscicida* have been reported. A large number of virulence factors/systems, such as type III (T3SS) and type VI (T6SS) secretion systems, the LuxS/AI-2 quorum sensing system, molecular chaperons, the RNA-binding protein Hfq, ferric uptake regulator (Fur), and lysozyme inhibitors, are known to be involved in *E. piscicida* stress resistance, host immune escape, and pathogenicity [25–31]. However, study of haem uptake and utilization by *E. piscicida* is extremely limited.

There is a speculative haem utilization operon in the *E. piscicida* genome; the first two proteins were annotated as ChuW/HutW and ChuX/HutX, and the third protein was annotated as an epimerase [32]. According to sequence homology comparison and other pathogenic bacterial sequence information, we named the third protein in this speculative haem utilization operon HutZ. In this study, we characterized HutZ in *E. piscicida* (named HutZ_Ep_), examined its expression profiles under different conditions, and analysed its role in adversity and infection. Our results provide the first insights into the biological function of *E. piscicida* HutZ.

## Materials and methods

### Bacteria and growth conditions

*Escherichia coli* BL21 (DE3) was purchased from TransGen (Beijing, China). *E. coli* S17-1λpir was purchased from Biomedal (Sevilla, Spain). *E. piscicida* TX01 was isolated from diseased fish [33]. Bacteria were cultured in Luria–Bertani broth (LB) at 37 °C (for *E. coli*) or 28 °C (for *E. piscicida*). Where indicated, chloramphenicol, tetracycline, and polymyxin B were supplemented at concentrations of 30 μg/mL, 15 μg/mL, and 100 μg/mL, respectively; 2,2′dipyridyl (Dp) was supplemented at concentrations of 60 μM, 100 μM, or 150 μM; and haem was supplemented at concentrations of 0.5 μM or 20 μM.

### Construction of the *hutZ*_*Ep*_ mutation and its complementation

The primers used in this study are listed in Table 1. To construct a *hutZ*_*Ep*_ knockout strain, TX01Δ*hutZ*, in-frame deletion of a 441 bp segment (residues 13 to 453) of *hutZ*_*Ep*_ was performed by overlap extension PCR as follows: the first overlap PCR was performed with the primer pair HutZF1/R1, the second overlap PCR was performed with the primer pair HutZF2/R2, and the fusion PCR was performed with the primer pair HutZF1/R2. The PCR products amplified by the primer pair HutZF1/R2 were inserted into the suicide plasmid pDM4 at the *Bgl*II site, resulting in pDMHutZ. S17-1λpir was transformed with pDMHutZ, and the transformants were conjugated with TX01 as described previously [34]. The transconjugants were selected on LB agar plates supplemented with 10% sucrose. One of the colonies that were resistant to sucrose and sensitive to chloramphenicol was analysed by PCR, and the PCR products were subjected to DNA sequencing to confirm in-frame deletion. This strain was named TX01Δ*hutZ*. To construct the complementary strain TX01Δ*hutZ*C, *hutZ*_*Ep*_ was amplified by PCR with the primers HutZF3/R3, and the following experimental operations were performed, as described previously [34].

**Table 1:** Primers used in this study

Primer name	Sequence (5′–3′)
HutZKOF1	GGATCCTTAGCGCTGGTGCACAC (*Bam*HI)
HuttZKOR1	TCCAGCAACCACGGCGTCATGCGCGC
HutZKOF2	CGCCGTGGTTGCTGGATGGCGAAGCC
HutZKOR2	GGATCCCAGCATTTCCGGCGCGGAT (*Bam*HI)
HutZF3	ACACATTGCACTGGTTGA
HutZR3	GTACGCTCTTGCGTCAGT
HutZRTF	GCAGAGCAGCGGTATGGACTTT
HutZRTR	TTCCATCAGGCGGTACATCCA
HutZF5	GAGCTCATGACGCCGTGGATC (*Sac*I)
HutZR5	AAGCTTGCGCACGGGGCGCTC (*Hin*dIII)
HutZF1	CATATGATGACGCCGTGGATC (*Nde*I)
HutZR1	CTCGAGGCGCACGGGGCGCTC (*Xho*I)
HutWXF	AGTGGCAATCCTGCGATTT
HutWXR	TGTTGATAAGCGTGGTGACA
HutXZF	CGTGTGGTTTATCAACCCTG
HutXZR	TGGGCGAGATAGTCATGACC
HutPF4	ATTTAAATGCCCGGACAGGCGCTGAT (*Swa*I)
HutPR4	ATTTAAATGGTAACTCTCCGTTAATACCTGA (*Swa*I)
FurF1	GGATCCATGACTGACAACAACACC (*Bam*HI)
FurR1	AAGCTTGGCCTTTTCGTCGTGCA (*Hin*dIII)

### Resistance to acidic stress and to non-immune fish serum

TX01, TX01Δ*hutZ* and TX01Δ*hutZ*C were cultured in LB medium to exponential phase. To determine acid tolerance, LB agar plates with pH = 7 or pH = 5 were streaked with the three bacteria. The plates were incubated at 28 °C for 48 h, and bacterial growth was examined. For quantitative analysis, three strains were cultured in LB medium with acid stress conditions for 24 h, and then the populations of cultivated bacteria were counted by dilution plating. The experiment was performed three times.

TX01, TX01Δ*hutZ* and TX01Δ*hutZ*C were cultured in LB medium to exponential phase. Then, the cells were washed with PBS and resuspended in PBS. Approximately 10^5^ bacterial cells were mixed with 50 μL of fish serum or PBS (control). After incubation with mild agitation at 23 °C for 60 min, the mixtures were serially diluted and plated in triplicate on LB agar plates. The plates were incubated at 28 °C for 48 h, and the colonies that appeared on the plates were enumerated. The survival rate was calculated as follows: [(number of serum-treated cells)/(number of control cells)] × 100%. The experiment was performed three times.

### Biofilm assay and motility assay

TX01 and TX01Δ*hutZ* were cultured in LB medium to exponential phase and diluted to 10^6^ CFU/mL. The diluted cells were transferred into a 96-well polystyrene plate (Nunc, Denmark) and incubated at 28 °C for 24 h without agitation. Then, the wells were washed gently five times with PBS. The attached cells were treated with Bouin fixative for 1 h and stained with 1% crystal violet solution for 20 min. After the treatment, unbound dye was removed by rinsing the plate several times with PBS. The plate was air dried. Bound dye was eluted in methanol, and the *A*_570_ of eluates was measured. The experiment was performed three times.

The observation of biofilms by confocal laser scanning microscopy (CLSM) was performed as described by Chan et al. [35]. Briefly, TX01 and TX01Δ*hutZ* were grown in LB medium on glass-bottom dishes for 24 h at 28 °C. The dishes were rinsed to remove non-adherent bacteria and then stained with a LIVE/DEAD BacLight bacterial viability kit L-13152 (Invitrogen-Molecular Probes, Carlsbad, CA, USA) for observation of biofilms. The staining procedure involved incubation for 15 min at room temperature in the dark. The biofilms were observed using a Leica TCS-SP2-AOBS-UV confocal laser scanning microscope equipped with an argon ion laser. The observation of biofilms was also performed with a stereoscopic fluorescence microscope as described by Hufnagel et al. [36]. Briefly, TX01 and TX01Δ*hutZ* were grown in LB medium to an OD_600_ of 0.6, washed twice in YESCA broth (10 g of casamino acids and 1 g of yeast extract/L) and spotted onto YESCA CR (50 μg/mL) medium for 48 h at 28 °C. The biofilms were observed by stereoscopic fluorescence microscopy.

To measure motility, TX01 and TX01Δ*hutZ* were cultured in LB medium to an OD_600_ of 1.0, and 2 μL of cell suspensions were spotted onto the centre of fresh swimming plates, which contained LB medium plus 0.3% (w/v) agar. The plates were then incubated at 28 °C. After 48 h, the motility of the bacteria was assessed by examining the diameter of the motility halo on the soft agar. The experiment was performed three times.

### Invasion of host cell lines

Examination of interactions between FG cells and *E. piscicida* was performed as described previously [37]. Briefly, FG cells were cultured in 96-well cell culture plates to a monolayer and mixed with the strain TX01 or TX01Δ*hutZ* at a multiplicity of infection (MOI) of 10:1. After incubation at 25 °C for 1 h and 2 h, the plates were washed three times with PBS. To determine the number of bacterial cells associated with the entire FG cell, the washed FG cells were lysed with 200 μL of 1% (vol/vol) Triton X-100 in PBS, and the number of bacteria was counted by dilution plating. To determine the numbers of bacterial cells that had penetrated into FG cells, the abovementioned washed FG cells were incubated with gentamicin (100 μg/mL) for 2 h to kill extracellular bacteria. After washing three times with PBS, the cells were incubated for 0 h to 8 h. FG cells were lysed and plated as described above.

### Fish and experimental challenges for bacterial dissemination in vivo

Clinically healthy Japanese flounder (*Paralichthys olivaceus*) (average 12.8 g) were purchased from a commercial fish farm of Shandong. The fish were maintained at ~22 °C in aerated seawater and fed daily with commercial dry pellets. Fish were acclimatized in the laboratory for 2 weeks. Before the experiment, fish were randomly sampled and examined for the presence of bacteria in the blood, liver, kidney, and spleen, and no bacteria were detected from the sampled fish, as described previously [38]. For tissue collection, fish were euthanized with an overdose of MS222 (tricaine methanesulfonate) (Sigma, USA). For tissue dissemination analysis, TX01, TX01Δ*hutZ*, and TX01Δ*hutZ*C were cultured in LB medium to an OD_600_ of 0.6. The cells were washed with PBS and resuspended in PBS to 10^6^ CFU/mL. Fish were divided randomly into four groups and infected by intraperitoneal injection with 50 μL of TX01, TX01Δ*hutZ*, TX01Δ*hutZ*C, or PBS. The kidney and spleen were taken aseptically from the fish at 24 h and 48 h post-infection (hpi). Bacterial recovery from the tissues was determined as reported previously [33]. The experiment was performed in triplicate.

### Reactive oxygen species (ROS) production

Flounder head kidney (HK) macrophages were prepared as described previously [39]. ROS production was determined as follows. Flounder HK macrophages in a 96-well microplate (~ 10^5^ cells/well) were incubated with TX01, TX01Δ*hutZ*, and TX01Δ*hutZ*C (10^6^ CFU/well) for 2 h. The plate was washed with PBS three times. One hundred microliters of 1 mg/mL nitroblue tetrazolium (Sangon, Shanghai, China) in L-15 was added to the cells. After incubation at 25 °C for 2 h, the reaction was stopped by adding 100% methanol. The plate was washed with 70% methanol, and reduced formazan was solubilized in 100 μL of 2 M KOH and 120 μL of dimethyl sulfoxide. The plate was read at 630 nm with a microplate reader. The experiment was performed three times.

### Quantitative real-time reverse transcriptase PCR (RT-qPCR) analysis of *hutZ*_*Ep*_ expression under different environmental conditions and in the *fur* mutant

To examine *hutZ*_*Ep*_ expression under in vitro conditions, TX01 was grown in LB medium with different pH values (pH 5 or 7) at 28 °C and incubated with or without non-immune fish serum. The bacteria were harvested by centrifugation, and total RNA was extracted with an HP Total RNA kit (Omega Bio-Tek, USA). The RNA was treated with DNase with a RNase-Free DNase Set kit (Omega Bio-Tek, USA). One microgram of total RNA was used for cDNA synthesis with Superscript II reverse transcriptase (Invitrogen, Carlsbad, CA, USA). RT-qPCR was carried out as reported previously [34]. The experiment was performed three times.

A *fur* mutant strain of *E. piscicida* was obtained in a previous study (data not published). The wild-type *E. piscicida* TX01 and *fur* mutant strains were cultured in LB medium to the early exponential phase. Then, bacteria were harvested, and total RNA was extracted. The expression of *hutZ*_*Ep*_ in the two strains was examined by RT-qPCR as described above.

### Protein expression and purification

To construct pEtHutZ and pEtFur, which express HutZ_Ep_ and FurZ_Ep_, respectively, the sequences of *hutZ*_*Ep*_ and *fur*_*Ep*_ were amplified by PCR with the primers HutZF5/R5 and FurF1/R1, and the PCR products were ligated into pET32a and pET28a-SUMO, respectively. Recombinant HutZ (rHutZ) and rFur were purified as described previously [37]. Preparation of polyclonal antibodies against rHutZ and immunoblot assays were performed as previously described [37]. Protease activity analysis of rHutZ was performed as reported by Kim et al. [20]. Hemin-binding activity of rHutZ was evaluated as reported by Uchida et al. [16].

### Transcriptional regulation of the promoter of *hutZ*_*Ep*_ by Fur

The speculative promoter of *hutZ*_*Ep*_ (the 283 bp of DNA upstream of the *hutWXZ* operon), P283, was cloned by the primers HutPF4/HutPR4 and inserted into the *Swa*I site of pSC11, a promoter probe plasmid [40], which resulted in pSZ283. pSZ283 was introduced into *E. coli* DH5α by transformation and cultured on X-Gal (5-bromo-4-chloro-3-indolyl-beta-d-galactopyranoside) plates. DH5α/pSZ283 was then transformed with pT (control) and pTFur, which expressed Fur and was constructed as described by Wang et al. [40], and cultured on X-gal plates. The transformants were subjected to a β-galactosidase assay [40].

An electrophoresis mobility shift assay (EMSA) was performed as reported previously [41]. Briefly, the DNA fragment of the speculative promoter was amplified by PCR and labelled with carboxyfluorescein (Sangon, China). The labelled DNA was mixed with rFur and incubated at 37 °C for 30 min in 20 μL of binding buffer (1 M Tris–HCl, pH 8.0; 5 M NaCl; 0.1 M MgCl_2_; 0.5 M EDTA; 1 M DTT; 80% glycerol) with or without a negative control DNA fragment (NCD), a fragment of the pT plasmid. The samples were then separated by electrophoresis in nondenaturing 8% polyacrylamide gels. For competition assays, unlabelled DNA fragments were added into the assay buffer.

### Statistical analysis

All statistical analyses were performed with SPSS 18.0 software (SPSS Inc., Chicago, IL, USA). Data were analysed with analysis of variance (ANOVA), and statistical significance was defined as *P* < 0.05.

## Results

### Characterization of the sequence of HutZ_Ep_

**Figure 1:**
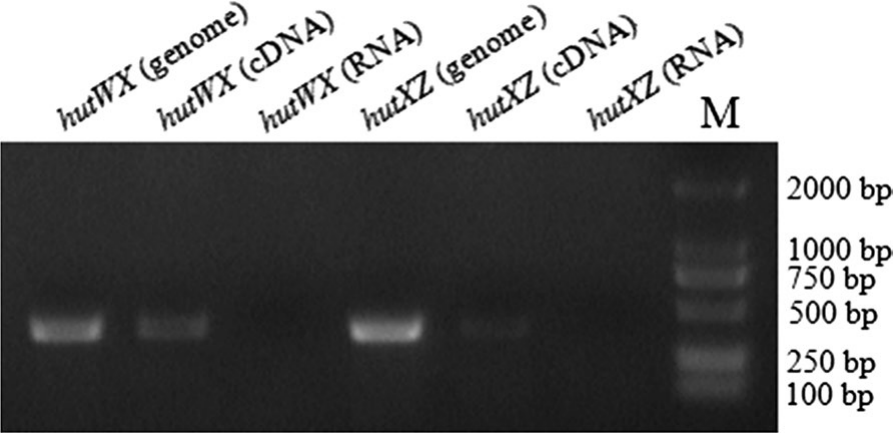
**The genes** ***hutW***_***Ep***_, ***hutX***_***Ep***_**, and** ***hutZ***_***Ep***_ **are co-transcribed.** Total RNA was isolated from *Edwardsiella piscicida* TX01 at a turbidity of 1.0 at 600 nm and treated with DNase I. cDNA was synthesized and used as the template in PCR. PCR products were amplified with specific primers for *hutW*_*Ep*_ and *hutX*_*Ep*_, *hutX*_*Ep*_ and *hutZ*_*Ep*_. Genomic DNA was used as a positive control, and total RNA was used as a negative control. M indicates a DNA ladder.

**Figure 2:**
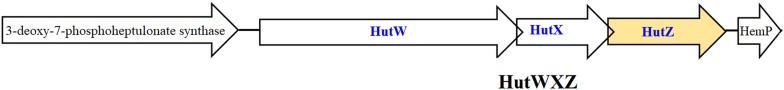
**Genetic organization of the HutZ and HutWXZ operon in** ***Edwardsiella piscicida***. *hutW*, *hutX*, and *hutZ* form the HutWXZ operon. The upstream gene of the HutWXZ operon is a 3-deoxy-7-phosphoheptulonate synthase, and the downstream gene is *hemP* [17]. Similar to in other species, in *E. piscicida*, the corresponding operon comprises only three genes. Therefore, we named the third protein epimerase HutZ, and the operon was named *HutWXZ*.

In a previous study of *E. piscicida*, we constructed a *fur* mutant strain, which exhibited much higher virulence than the wild-type *E. piscicida* strain TX01 (data not shown). Proteomic analysis showed that the expression of a protein annotated as an epimerase was significantly upregulated in the *fur* mutant strain compared to that in the wild-type strain (data not shown). Bioinformatics analysis showed that the epimerase may be part of an operon with two other proteins. To confirm this hypothesis, RT-PCR was performed, and the results showed that the three genes were co-transcribed (Figure 1). The first two proteins are homologues of the haem anaerobic degradation radical SAM methyltransferase ChuW/HutW and the haem utilization cytosolic carrier protein ChuX/HutX, respectively. In *E. coli*, the *Chu* operon consists of *chuS*, *chuW*, *chuX*, *chuY*, *chuU*, and *hmuV* [21]. In *V. cholerae*, the *Hut* operon contains only three genes, *hutW*, *hutX*, and *hutZ* [17]. Similar to the latter, in *E. piscicida*, the corresponding operon comprises only three genes. Therefore, we named the third protein epimerase HutZ, and the operon was named *HutWXZ* (Figure 2). HutZ_Ep_ shares moderate homology (50% identity) with *E. coli* ChuY. However, multiple conserved amino acids in ChuY and its homologues did not appear in HutZ_Ep_, including some important residues buried within the ChuY dimer interface [42], such as Glu94, Gln126, Thr132, Ser136, and Thr140 (Additional file 1). Furthermore, the spatial structure of HutZ_Ep_ is also different from that of ChuY (Additional file 1); for example, seven α-helices exist in HutZ_Ep_, but only six α-helices exist in ChuY [20].

**Figure 3:**
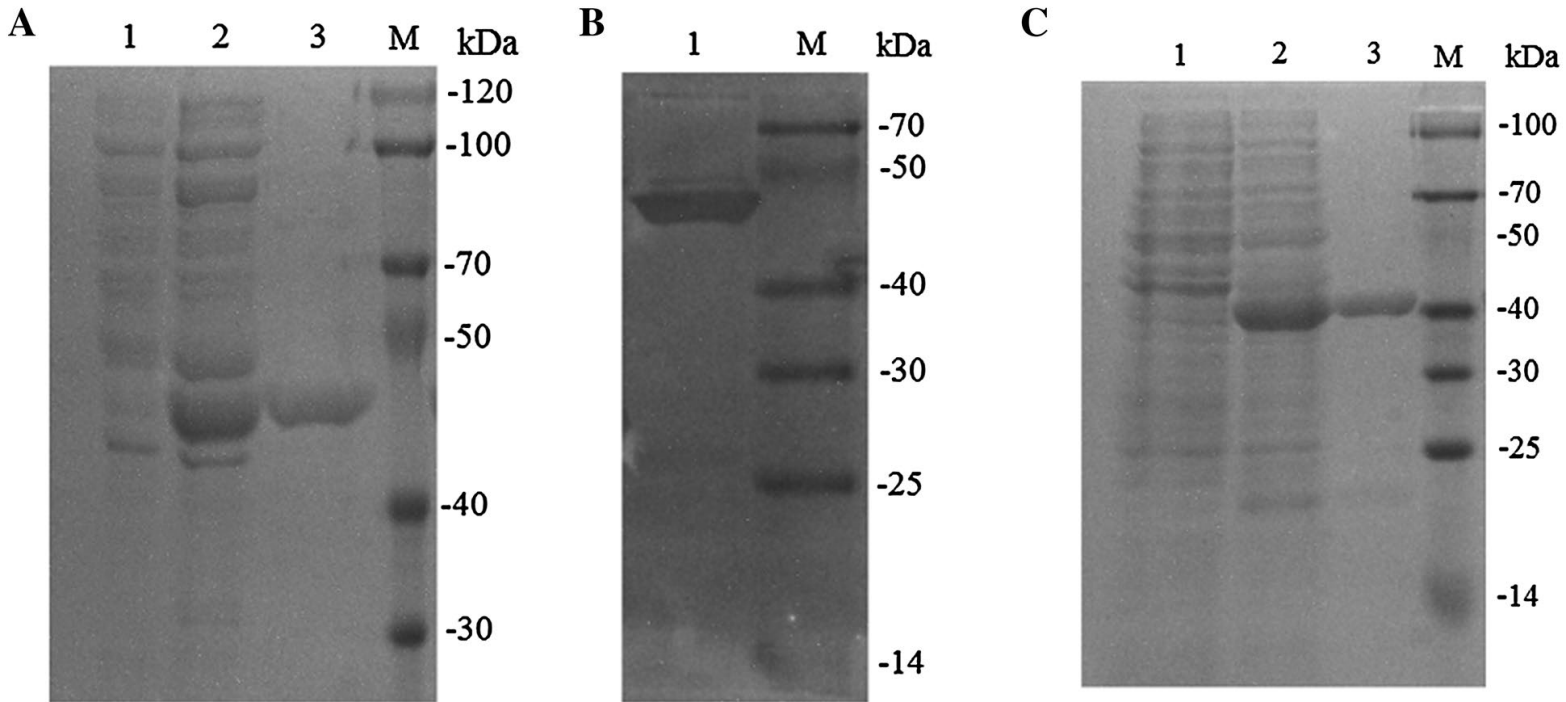
**SDS-PAGE analysis of recombinant HutZ and rFur. A** Whole-cell proteins were prepared from *E. coli* BL21(DE3)/pEtHutZ cultured in LB medium before (lane 1) and after (lane 2) IPTG induction. Lane 3, recombinant SoFer1 after purification by affinity chromatography. **B** Western immunoblot analysis of purified rHutZ. **C** Expression and purification of rFur.

To determine the function of HutZ_Ep_, the coding sequences of *hutZ*_*Ep*_ were expressed in and purified from *E. coli*. SDS-PAGE analysis showed that the purified protein exhibited a molecular mass comparable to that predicted for rHutZ (~ 48 kDa), and the purified protein was confirmed by western immunoblot analysis (Figure 3). Protease activity analysis based on the *A*_340_ showed that rHutZ had no obvious flavin reductase activity (data not shown). Based on UV–Vis spectroscopy, we examined the hemin-binding activity of rHutZ_Ep_, and the results showed that rHutZ_Ep_ did not exhibit obvious hemin-binding activity (data not shown). These results suggested that HutZ_Ep_ is probably not related to hemin utilization.

### Construction of an *E. piscicida hutZ* mutant

To examine its functional importance, the *hutZ* gene of *E. piscicida* TX01 was knocked out by markerless in-frame deletion of the region encoding the amino acid residues 13 to 453. The resulting mutant was named TX01Δ*hutZ*.

### HutZ_Ep_ is not required for iron acquisition and haem utilization

**Figure 4:**
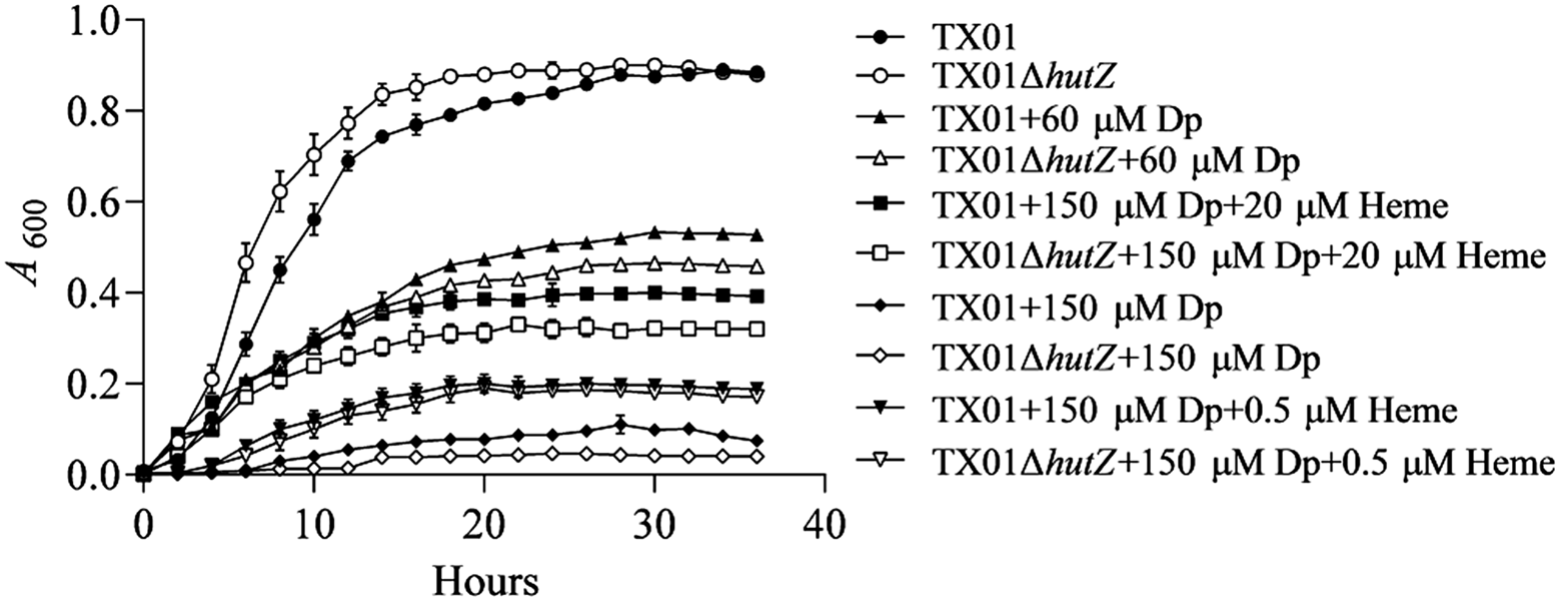
**Growth analysis of TX01 and TX01Δ*hutZ***. TX01 and TX01Δ*hutZ* were cultured in LB medium, in LB medium supplemented with 2,2′dipyridyl (Dp), or in LB medium with Dp and haem, and the cell density was measured at different time points by determining the absorbance at OD_600_. Data are presented as the means ± SEMs (*N* = 3).

Growth analysis showed that when cultured in LB medium, TX01Δ*hutZ* exhibited a slightly faster generation time than TX01 at the logarithmic phase but reached cell densities similar to those of TX01 at the stationary phase (Figure 4). When cultured under conditions of iron depletion (with 60 µM Dp), the growth of both TX01Δ*hutZ* and TX01 was retarded and exhibited a similar growth rate, although TX01Δ*hutZ* displayed a slightly slower growth rate than TX01. When the concentration of Dp was increased to 150 µM, both TX01Δ*hutZ* and TX01 were barely able to grow (Figure 4). To determine the expression of *hutZ*_*Ep*_ under normal conditions (i.e., cultured in LB medium) and iron deficiency conditions (i.e., cultured in LB medium with 100 µM Dp), RT-qPCR was performed, and the results showed that the expression of *hutZ*_*Ep*_ remained unchanged when bacteria faced iron deficiency compared to the expression of *hutZ*_*Ep*_ under normal conditions (data not shown). To examine whether *hutZ*_*Ep*_ is a key factor involved in haem utilization, strains were grown in iron deficiency medium (with 150 µM Dp) supplemented with a low concentration of haem (0.5 µM) or high concentration of haem (20 µM), and strain growth was surveyed. The results showed that with the increase in haem concentration, growth of both TX01Δ*hutZ* and TX01 was improved and exhibited a similar trend with no significant difference (Figure 4). These results, combined with the aforementioned results, showed that HutZ_Ep_ is not required for iron acquirement and haem utilization.

### Effect of *hutZ*_*Ep*_ mutation on bacterial resistance against acid stress

**Figure 5:**
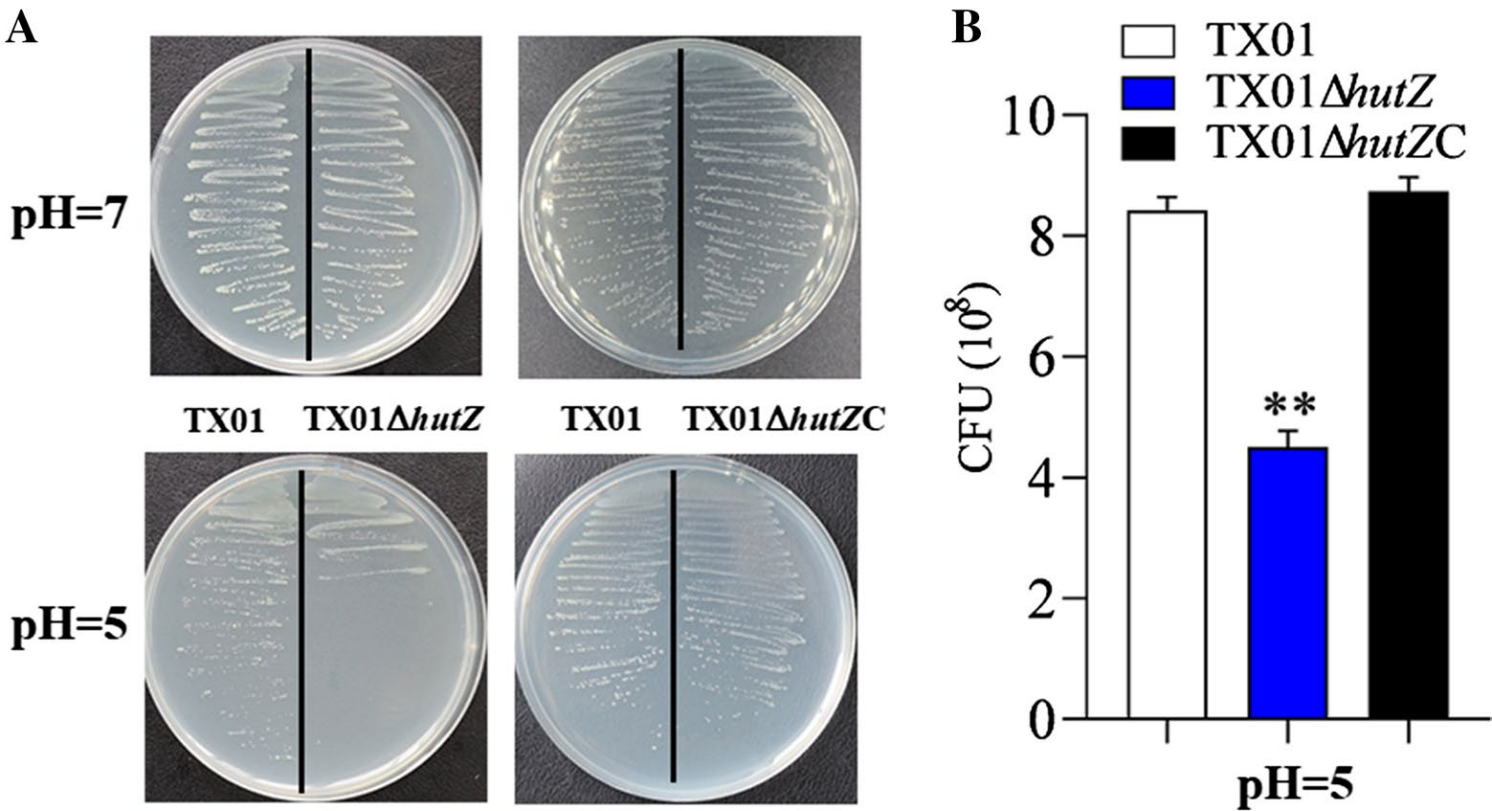
**Sensitivity of** ***Edwardsiella piscicida*** **to acid stress. A** TX01, TX01Δ*hutZ*, and TX01Δ*hutZ*C were cultured in LB medium and on LB agar plates at pH = 7 and pH = 5 at 28 °C for 24–48 h. **B** Bacteria cultured to logarithmic stage were transferred to LB medium at pH = 5, and the populations of cultivated bacteria were counted by dilution plating. Data are the means of three independent experiments and are presented as the means ± SEMs (*N* = 3). N, the number of times the experiment was performed. **P < 0.01.

Since the *fur* mutant caused an increase in the virulence of *E. piscicida* and enhanced the expression of *hutZ*_*Ep*_, we speculated that HutZ_Ep_ participated in the stress resistance and pathogenicity of *E. piscicida* and detected the acid tolerance of the TX01Δ*hutZ* mutant. Growth analysis showed that when cultured on LB agar medium, TX01Δ*hutZ* and TX01 exhibited a comparable growth rate, in line with the result in LB medium. When cultured under acidic conditions, TX01Δ*hutZ* grew more poorly than TX01, and the survival of TX01Δ*hutZ* was significantly lower than that of TX01 (Figure 5). *hutZ*_*Ep*_ expression was analysed under normal conditions and acid stress by RT-qPCR, and the results showed that the expression of *hutZ*_*Ep*_ was unchanged when bacteria faced acid stress compared to the expression of *hutZ*_*Ep*_ under normal conditions (data not shown).

### Effect on bacterial resistance to non-immune fish serum

**Figure 6:**
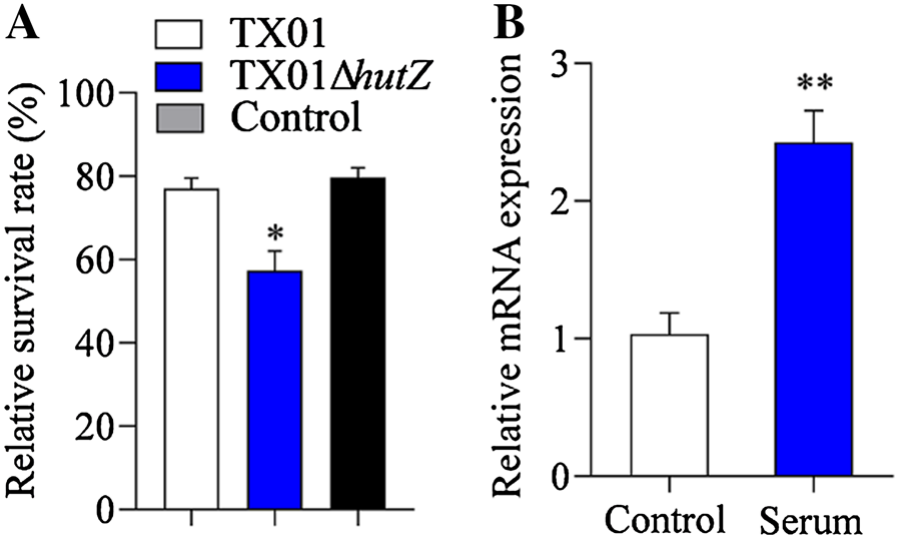
**Effects of** ***hutZ***_***Ep***_ **mutation on resistance to serum and** ***hutZ***_***Ep***_ **expression under serum tolerance. A** Survival of *E. piscicida* in fish serum. TX01, TX01Δ*hutZ*, and TX01Δ*hutZ*C were incubated with non-immune flounder serum or PBS (control). After incubation, the survival of the bacteria was determined by plate counting. **B** RT-qPCR was performed with total RNA extracted from cultured *Edwardsiella piscicida* incubated in LB medium and incubated in flounder serum. Data are presented as the means ± SEMs (*N* = 3). N, the number of times the experiment was performed. *P < 0.05; **P < 0.01.

To examine whether the *hutZ*_*Ep*_ mutation affected serum tolerance, TX01 and TX01Δ*hutZ* were incubated with non-immune flounder serum for 1 h, and the survival of bacteria was determined by plate counting. The results showed that TX01 exhibited apparent serum resistance, as 77% of cells survived after incubation with flounder serum. However, only 57.3% of TX01Δ*hutZ* cells survived after serum treatment, which was significantly lower than that for TX01 (Figure 6A). The expression of *hutZ*_*Ep*_ was also analysed under normal conditions and serum stress by RT-qPCR, and the result showed that the expression of *hutZ*_*Ep*_ was significantly enhanced when bacteria faced serum stress compared to the expression of *hutZ*_*Ep*_ under normal conditions (Figure 6B).

### Effect of *hutZ*_*Ep*_ mutation on biofilm formation and motility

**Figure 7:**
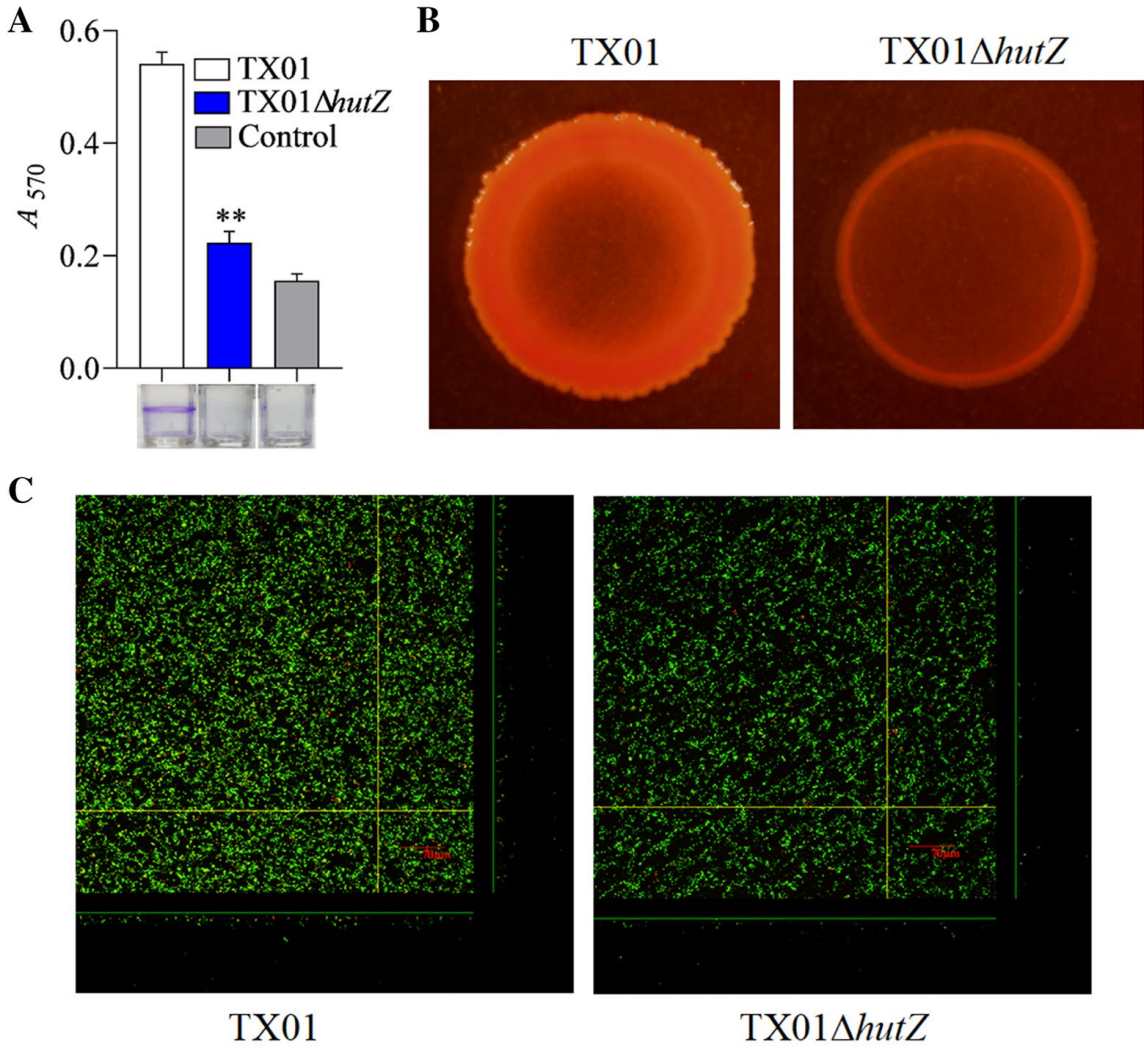
**Effects of** ***hutZ***_***Ep***_ **mutation on biofilm growth. A** Biofilm-forming capacity of *E. piscicida*. TX01 and TX01Δ*hutZ* were incubated in polystyrene plates, and biofilm formation was determined by measuring the *A*_570_ of the final eluates. **B** The viability of biofilm growth of *E. piscicida* as determined by confocal laser scanning microscopy (CLSM). Cells in the biofilms were stained with a BacLight LIVE/DEAD kit to reveal viable (green fluorescence) and non-viable (red fluorescence) bacteria. Data are presented as the means ± SEMs (*N* = 3). N, the number of times the experiment was performed. **P < 0.01.

Next, we surveyed whether HutZ has any relation with biofilm formation. TX01 and TX01Δ*hutZ* were cultured in polystyrene plates. After treating with Bouin fixative and crystal violet, biofilm formation was assayed. The results showed that the biofilm growth of TX01Δ*hutZ* was significantly slower than that of TX01 and was comparable to that of the control (LB medium without bacteria) (Figure 7A). Meanwhile, we surveyed the two strains’ biofilm growth on YESCA agar, and the results showed that the biofilm formation capability of TX01Δ*hutZ* was markedly weaker than that of TX01 (Figure 7B). We next acquired images of the biofilms of the strains TX01 and TX01Δ*hutZ* using confocal laser scanning microscopy (CLSM). The results showed that deletion of *hutZ*_*Ep*_ led to a substantial decrease in the thickness and density of the biofilm during biofilm formation compared to those of the parental strain (Figure 7C). To explore whether *hutZ*_*Ep*_ was directly related to *E. piscicida* biofilm formation, the expression of several biofilm-related genes, such as *bsmA*, *bssS*, *hmsP*, and *csgD* [43], was investigated by RT-qPCR, and the results showed that the expression of these biofilm-related genes remained unchanged when *hutZ*_*Ep*_ was deleted (data not shown). These findings indicated that HutZ_Ep_ directly participates in biofilm growth and is probably a novel biofilm-related factor.

**Figure 8:**
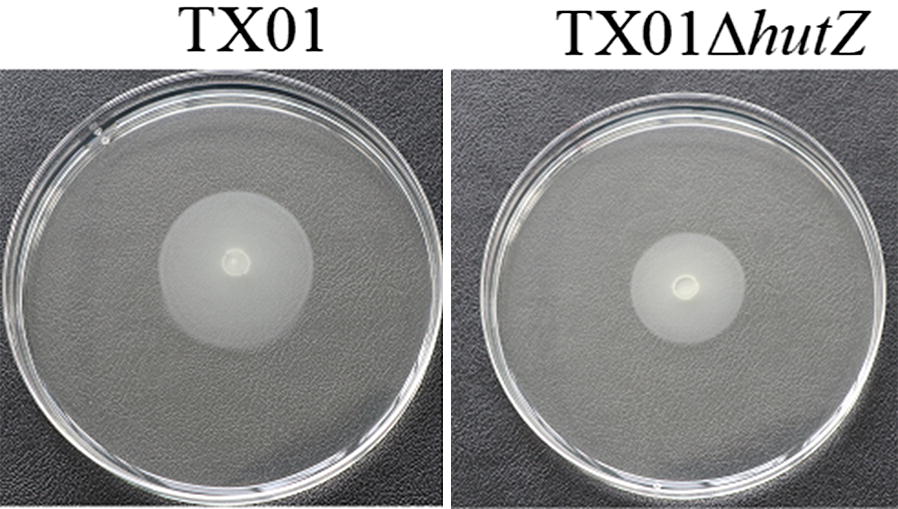
**Effects of** ***hutZ***_***Ep***_ **mutation on motility.** TX01 and TX01Δ*hutZ* were cultured in LB medium to an OD600 of 1.0, and 5 μL of cell suspensions were spotted onto the centre of swimming plates containing LB medium plus 0.3% (w/v) agar. The plates were incubated at 28 °C for 2 days.

To investigate whether deletion of *hutZ*_*Ep*_ has any effect on bacterial motility, TX01 and TX01Δ*hutZ* were dripped on soft LB agar plates. After culturing for 24 h, the mobility was examined, and the results showed that the motility zone diameter of TX01Δ*hutZ* was smaller (average diameter 25 ± 1.2 mm) than that of TX01 (average diameter 33 ± 1 mm) (Figure 8). These findings indicated that *hutZ*_*Ep*_ played an essential role in biofilm formation and motility.

### Effect of *hutZ*_*Ep*_ mutation on pathogenicity

**Figure 9:**
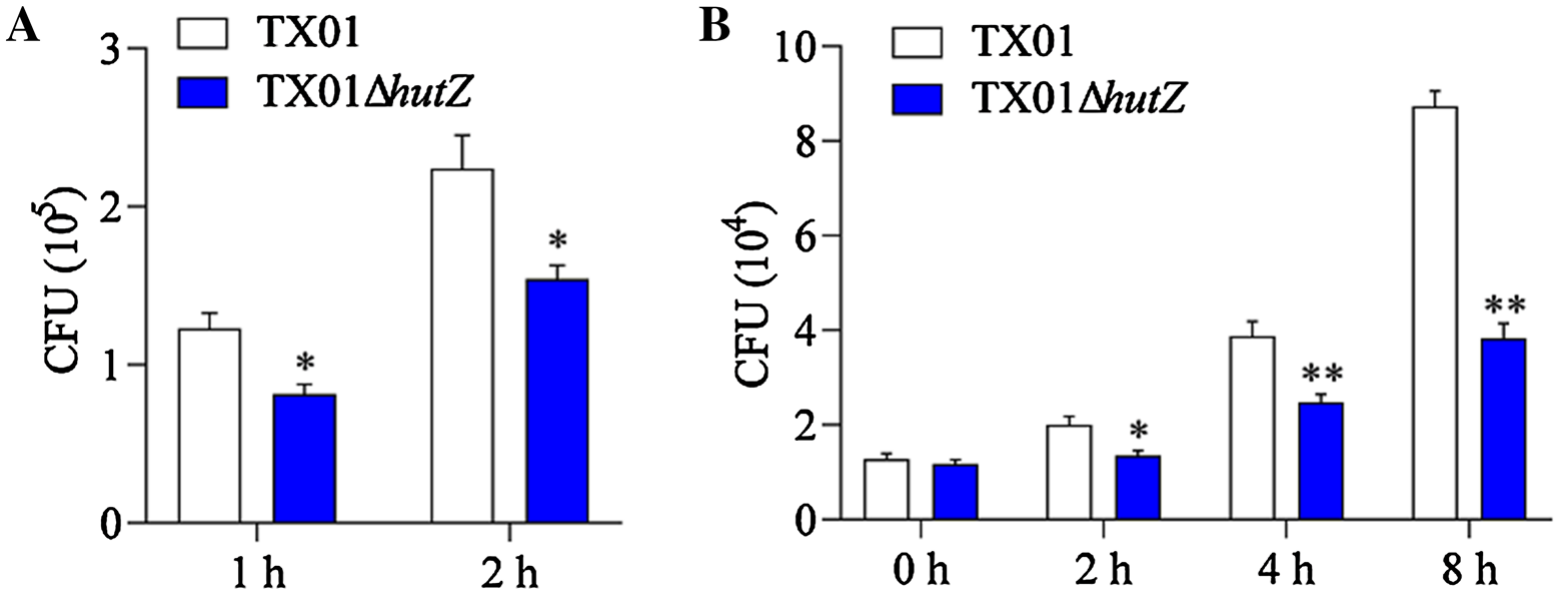
**Effect of** ***hutZ***_***Ep***_ **mutation on cellular infection and replication. A** *Edwardsiella piscicida* invasion of flounder gill cells (FG cells). FG cells were infected with the same dose of TX01 and TX01Δ*hutZ* for various amounts of time and washed with PBS. Then, FG cells were lysed, and the bacteria associated with and invaded into the host cells were enumerated. **B** Replication of *E. piscicida* in FG cells. After infecting with TX01 and TX01Δ*hutZ* for 2 h, FG cells were treated with gentamicin for 2 h. The cells were then incubated further for various amounts of time, and the number of intracellular bacteria was determined by plate counting. Data are the means of three independent experiments and are presented as the means ± SEMs (*N* = 3). N, the number of times the experiment was performed. *P < 0.05, **P < 0.01.

**Figure 10:**
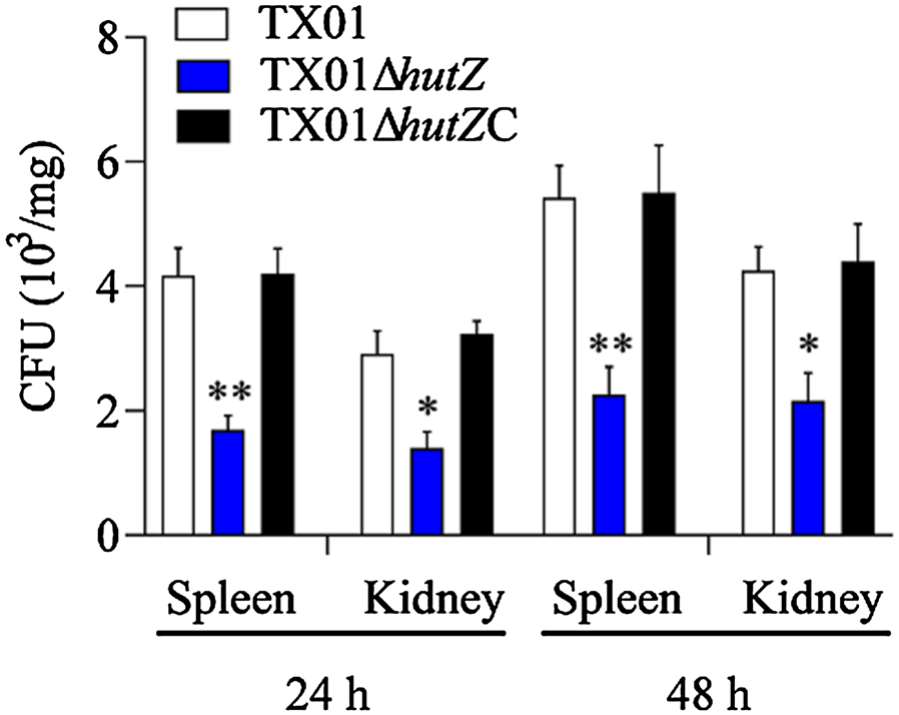
**Bacterial dissemination in fish tissues.** Flounders were infected with the same dose of TX01, TX01Δ*hutZ*, or TX01Δ*hutZ*C, and bacterial recovery from the spleen and kidney was determined by plate counting at 24 h and 48 hpi. Data are presented as the means ± SEMs (*N* = 3). N, the number of times the experiment was performed. *P < 0.05, **P < 0.01.

Since deletion of *hutZ*_*Ep*_ has an effect on bacterial resistance to serum and biofilm formation and the physiological role of *hutZ*_*Ep*_ has not yet been identified, we assessed the role of *hutZ*_*Ep*_ in *E. piscicida* pathogenesis in in vitro and in vivo infection experiments. To examine whether HutZ_Ep_ played any role in interaction with host cells, cultured FG cells were incubated with TX01 or TX01Δ*hutZ*, and the bacterial cells associated with the host cells were enumerated. The results showed that the amount of TX01Δ*hutZ* recovered from the entire (i.e., from the surface and the intracellular milieu) FG cell culture was significantly lower than that of TX01 after infecting for 1 h and 2 h (Figure 9A). It is known that *E. piscicida* is able to survive and replicate in host cells [29]. To examine whether the *hutZ*_*Ep*_ mutation played any role in the intracellular survival of TX01, FG cells were incubated with *E. piscicida*, and extracellular bacteria were killed. The cells were then incubated further for various amounts of time, and the number of intracellular bacteria was determined by plate counting. The results showed that the number of intracellular TX01Δ*hutZ* recovered from the cells was significantly lower than that of TX01 at various time points (Figure 9B). Hence, the *hutZ*_*Ep*_ mutation significantly impaired the ability of *E. piscicida* to adhere to and invade host cells. To examine the effect of the *hutZ*_*Ep*_ mutation on tissue infectivity, flounder were infected with the same dose of TX01 or TX01Δ*hutZ*, and bacterial recovery from the spleen and kidney was determined at 24 and 48 hpi. The results showed that bacterial recovery from TX01Δ*hutZ*-infected fish was significantly lower than that from TX01-infected fish at 24 hpi and 48 hpi (Figure 10).

### Effect of *hutZ*_*Ep*_ mutation on resistance against the immune response of host macrophages

**Figure 11:**
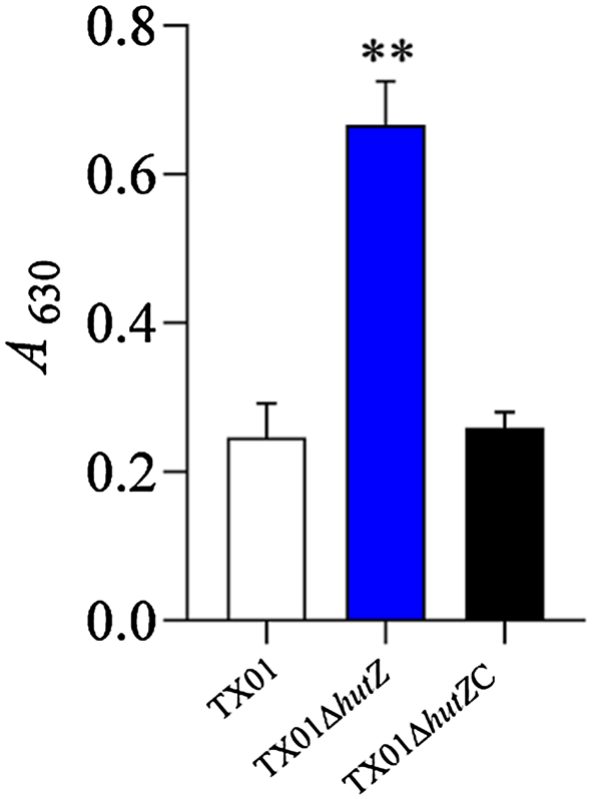
**Effect of** ***hutZ***_***Ep***_ **mutation on the immune response of macrophages.** Flounder head kidney macrophages were infected with TX01, TX01Δ*hutZ*, or TX01Δ*hutZ*C, and reactive oxygen species production in the cells was determined at 2 hpi. Data are presented as the means ± SEMs (*N* = 3). **P < 0.01.

Since TX01Δ*hutZ* exhibited attenuated infectivity in the host, we wanted to examine whether the *hutZ*_*Ep*_ mutation affected the ability of *E. piscicida* to block the activation of host phagocytes. For this purpose, flounder HK macrophages were infected with TX01 or TX01Δ*hutZ*, and the cellular production of ROS was determined. The results showed that ROS levels in TX01Δ*hutZ*-infected cells were significantly higher than those in TX01-infected cells (Figure 11).

### Genetic complementation of the *hutZ*_*Ep*_ deletion and its effect on virulence

To examine whether the stress resistance and virulence defect observed for TX01Δ*hutZ* were indeed due to the *hutZ*_*Ep*_ deletion, the strain TX01Δ*hutZ*C was created, which is a genetic variant of TX01Δ*hutZ* that expresses *hutZ*_*Ep*_ *in trans* from a plasmid. In contrast to TX01Δ*hutZ*, TX01Δ*hutZ*C exhibited a comparable resistance against acid stress and non-immune fish serum to those of TX01 (Figures 5 and 6). Following infection of flounder HK macrophages, TX01Δ*hutZ*C-induced production of ROS was similar to that induced by TX01 infection (Figure 11). Likewise, the bacterial dissemination capacity of TX01Δ*hutZ*C in fish tissues was comparable to that of TX01 (Figure 10).

### Expression of *hutZ*_*Ep*_ is regulated by Fur (ferric uptake regulator)

**Figure 12:**
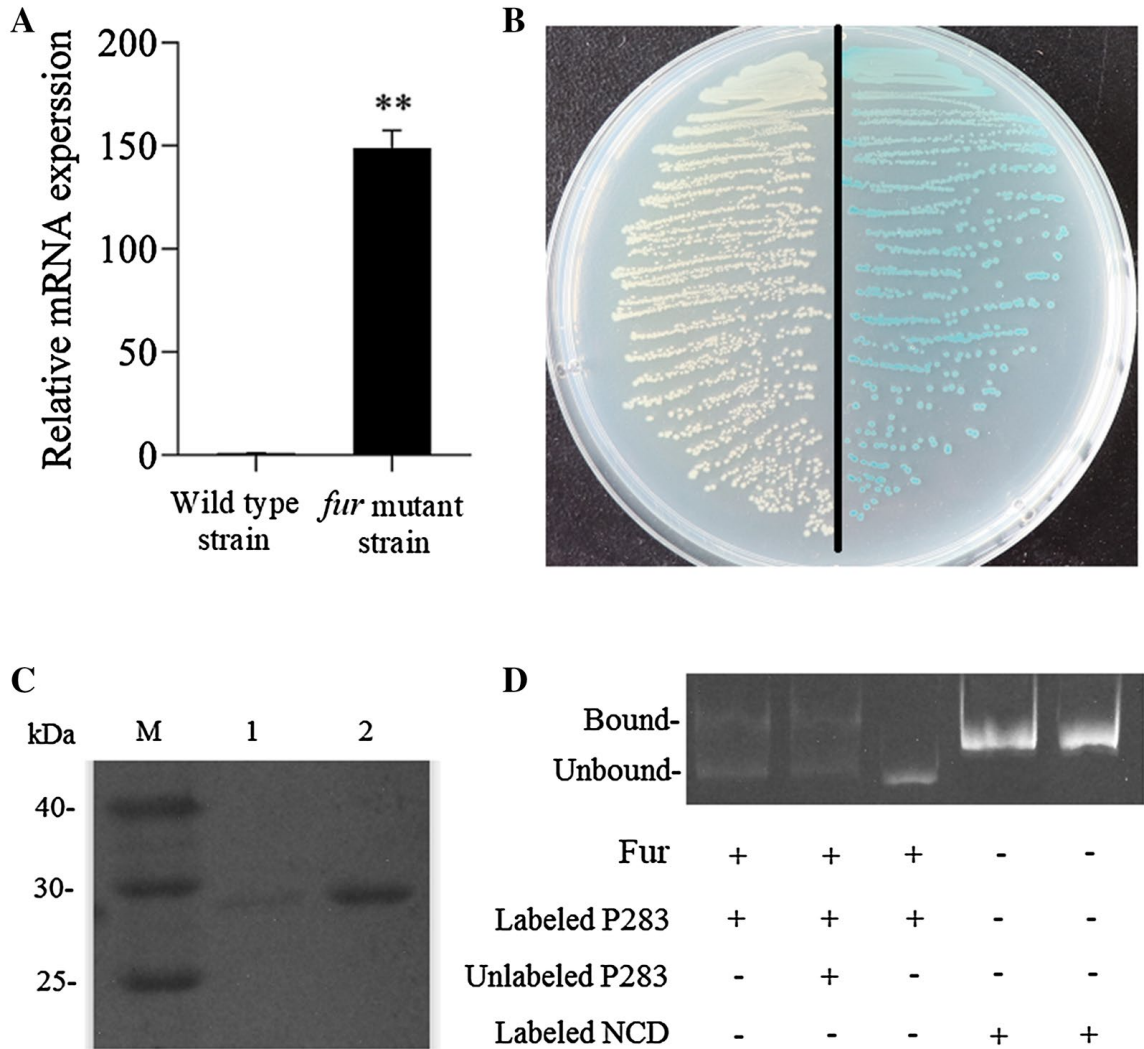
**Expression of** ***hutZ***_***Ep***_ **is regulated by Fur. A** RT-qPCR was performed with total RNA extracted from wild-type TX01 and a *fur* mutant strain cultured in normal LB medium. The expression level of *hutZ*_*Ep*_ in the wild-type TX01 strain was set at 1. **B** DH5α/pSZ283/pTFur and DH5α/pSZ283/pT were streaked and cultured on LB plates with X-gal, kanamycin, and ampicillin. **C** The expression of HutZ was examined by Western blot. 1. the expression of HutZ in wild type TX01; 2. the expression of HutZ in fur mutant strain. **D** Interaction between Fur and the speculative promoter regions of *hutZ*_*Ep*_. An electrophoresis mobility shift assay (EMSA) was performed in binding buffer containing Fur, unlabelled or carboxyfluorescein-labelled negative control DNA (NCD), and carboxyfluorescein-labelled D333. The negative control DNA (NCD) was derived from a fragment of the pT plasmid. Data are presented as the means ± SEMs (*N* = 3). N, the number of times the experiment was performed. **P < 0.01.

As mentioned above, HutZ expression was significantly upregulated in the *fur* mutant strain by proteomic analysis, so we detected the expression of *hutZ*_*Ep*_ at the mRNA and protein levels. RT-qPCR showed that the expression of *hutZ*_*Ep*_ in the *fur* mutant strain was 145-fold higher than that of *hutZ*_*Ep*_ in the wild-type strain (Figure 12A). Western blotting showed that the expression of HutZ_Ep_ in the *fur* mutant was also significantly higher than that of HutZ_Ep_ in the wild-type strain (Figure 12C). To detect the regulatory effect of Fur on the promoter activity of *hutZ*_*Ep*_, the speculative promoter of *hutZ*_*Ep*_, P283, was cloned into the promoter probe plasmid pSC11, resulting in DH5α/pSZ283. When DH5α/pSZ283 was cultured on LB agar plates with X-gal, the bacterial colonies were blue, which indicated that P283 has promoter activity. DH5α/pSZ283 was then transformed with pTFur (expresses Fur) and pT (control). On an X-gal plate, the blue of DH5α/pSZ283/pTFur was obviously weak compared with that of DH5α/pSZ283/pT (Figure 12B). β-galactosidase assays showed that Miller units produced by DH5α/pSZ283/pTFur (2.11 ± 0.15) were significantly lower than those produced by DH5α/pSZ283/pT (201.12 ± 0.10). These results indicated that Fur negatively regulated the transcription of *hutZ*_*Ep*_. To further analyse the function of Fur, rFur was expressed and purified from *E. coli* (Figure 3). An electrophoresis mobility shift assay (EMSA) showed that the purified rFur could bind the speculative promoter P283 (Figure 12D), which indicated that HutZ is directly regulated by Fur.

## Discussion

Haem utilization systems play important roles in bacterial iron acquisition, adversity adaptation and pathogenicity. To date, there are no reports about haem utilization in *E. piscicida*. In this study, a speculative haem utilization protein, HutZ_Ep_, was characterized in *E. piscicida*. HutZ_Ep_ is encoded along with two other proteins. The first two other proteins were annotated as haem anaerobic degradation radical SAM methyltransferase ChuW/HutW and haem utilization cytosolic carrier protein ChuX/HutX, respectively, in the genome [32]. In *E. coli*, the *chu* gene cluster contains several genes, such as *chuS*, *chuW*, *chuX*, *chuY*, *chuU*, and *hmuV*, which form an operon and are involved in haem/iron acquisition and homeostasis. [21]. A similar operon also exists in *Shigella dysenteriae* [44]. However, in *V. cholerae*, the haem utilization operon contains only three genes, *hutW*, *hutX*, and *hutZ* [17]. Similarly, the *hugWXZ* operon was found in *P. shigelloides* [18]. In *E. piscicida*, we named the third gene *hutZ*_*Ep*_, and the operon was called *hutWXZ*_*Ep*_.

Since *E. piscicida* is a member of Enterobacteriales, we wanted to determine whether HutZ_Ep_ has a function similar to that of ChuY. ChuY catalyses FMN reduction using NADPH or NADH as the electron donor, and ChuY also possesses hemin-binding activity [20]. However, unlike ChuY, we did not find that rHutZ exhibited obvious flavin reductase activity and hemin-binding activity, which suggested that HutZ_Ep_ is probably not related to hemin utilization. Differences in operon composition, conserved residues, and structure perhaps lead to differences in functionality between HutZ_Ep_ and ChuY. Moreover, deletion of *hutZ*_*Ep*_ had no significant effect on the growth of *E. piscicida* under iron deficiency conditions. It has been reported that HutZ in *V. cholerae* is a cytoplasmic haem-binding protein and is required for efficient haem degradation or haem utilization [15, 16, 45, 46]. HugZ from *P. shigelloides* was needed for survival when haem was used as an iron source [18]. However, our results showed that *hutZ*_*Ep*_ is not involved in haem utilization. These results, combined with the aforementioned results, showed that HutZ_Ep_ is not required for iron acquisition and haem utilization.

Since HutZ_Ep_ is irrelevant to iron acquisition, we wanted to determine whether it possesses other functions, especially adversity resistance and pathogenicity functions. Acid tolerance is an important trait for various pathogens during infection and is regulated by the regulator Fur in a variety of pathogens, such as *Salmonella typhimurium*, *E. coli*, and *Aeromonas salmonicida* [47–49]. We found that the deletion of *hutZ*_*Ep*_ markedly attenuated the acid tolerance capability of *E. piscicida*. For *E. piscicida*, evasion of serum-mediated bactericidal activity is a characteristic phenotype, but the mechanism is still poorly understood. It has been reported that *E. piscicida* evades serum killing by preventing complement activation via the alternative pathway [50]. Chen et al. [28] found that *E. piscicida* tunes the tricarboxylic acid cycle to evade complement-mediated killing, which reveals a previously unknown membrane potential-dependent mechanism of serum resistance. Two novel serum-induced proteins, Sip1 and Sip2, were found to be essential to serum resistance, which are also different from known mechanisms [29, 51]. Other virulence factors involved in resistance against the bactericidal effect of hos serum include the serine protease autotransporter Tsh, lysozyme inhibitor Ivy, and thioredoxin TrxH [34, 39, 52]. In this study, deletion of *hutZ*_*Ep*_ decreased the resistance of *E. piscicida* against host serum killing, which indicated that it is a novel virulence factor related to serum resistance. However, its mechanism requires further investigation.

Most bacteria can switch between a planktonic form and a biofilm mode, which aids in bacterial adaptation to environmental signals and stresses. Gram-negative bacteria, such as *E. coli*, form biofilms that consist of a bacterial colony embedded in a matrix of extracellular polymeric substances that protect the microbes from adverse environmental conditions and result in infection [53]. In *E. piscicida*, a number of virulence factors have been found to be relevant to biofilm formation. Among these factors, some inhibit biofilm formation. For example, the type III translocon protein EseC inhibits biofilm formation by sequestering the regulator EseE [54], and an *rpoS* sigma factor mutant displayed markedly increased biofilm formation [55]. Deletion of the *ugd* gene, which encodes UDP-glucose dehydrogenase, enhanced autoaggregation and biofilm formation [56]. However, additional genes are essential for biofilm formation by *E. piscicida*. EseB is a prerequisite for autoaggregation and biofilm formation [57]. Deficiency in multiple genes, such as the serine protease autotransporter *tsh*, *rcsB*, the sigma factor *rpoN*, the invasin gene, the flagellar genes *fliC*, *flhDC*, and the quorum sensing-related gene *luxS*, results in markedly decreased biofilm formation [33, 34, 58–62]. In the current study, the biofilm formation ability of the *hutZ*_*Ep*_ mutant strain TX01Δ*hutZ* was markedly weaker than that of the wild-type strain TX01. The expression of some known biofilm-related genes was not affected by *hutZ*_*Ep*_. These findings indicated that HutZ_Ep_ directly participates in biofilm growth and is probably a novel biofilm-related factor.

Bacterial biofilm formation is often closely related to motility. For example, RpoX plays distinct roles in stress response, motility, and biofilm formation in the marine pathogen *Vibrio alginolyticus* [63]. ToxR is required for the biofilm formation and motility of *Vibrio parahaemolyticus* [64]. Flagellar genes affect both bacterial motility and biofilm formation [61]. In accordance with these reports, our study showed that HutZ_Ep_ was involved in the motility of *E. piscicida*.

These findings clearly demonstrated that *hutZ*_*Ep*_ played an essential role in adversity resistance, biofilm formation, and motility, which indicated that *hutZ*_*Ep*_ was most likely involved in pathogenicity. Therefore, we examined the effect of *hutZ*_*Ep*_ on *E. piscicida* pathogenicity. The results showed that inactivation of *hutZ*_*Ep*_ significantly weakened the ability of *E. piscicida* to invade host cells. Similarly, the capability of *E. piscicida* to survive and replicate in host cells significantly declined when *hutZ*_*Ep*_ was inactivated. Moreover, an in vivo experiment showed that TX01Δ*hutZ* had a severely reduced ability to infect host tissues. In support of these results, the host immune response induced by TX01 and TX01Δ*hutZ* was examined, and the results showed that reactive oxygen species (ROS) levels in TX01Δ*hutZ*-infected macrophages were significantly higher than those in TX01-infected cells. Introduction of an *in trans*-expressed *hutZ*_*Ep*_ gene restored the lost virulence of TX01Δ*hutZ*. These findings indicate that *hutZ*_*Ep*_ is vital to the pathogenicity of *E. piscicida*.

The abovementioned results showed that *hutZ*_*Ep*_ plays a role in resistance against acid stress, but the expression of *hutZ*_*Ep*_ did not change under low pH conditions. *hutZ*_*Ep*_ also plays a role in resistance against non-immune fish serum. However, the expression of *hutZ*_*Ep*_ was significantly enhanced when bacteria faced serum stress. These results suggest that there may be a complicated relation between the expression and function of *hutZ*_*Ep*_. In *V. cholerae*, HutZ is required for efficient haem utilization, and its promoter region contains several potential binding sites for the iron regulatory protein Fur [16]. Moreover, the synthesis of HutZ is negatively regulated by iron [15]. Haem uptake or utilization operon is frequently regulated by Fur [65]. Fur was initially considered a regulator of genes associated with iron uptake. With in-depth research, it is clear that Fur is a global regulator and is involved in a variety of cellular processes, including stress response and virulence [66]. In our study, we confirmed that HutZ_Ep_ was directly regulated by Fur. Although HutZ was not required for iron acquisition and haem utilization, HutZ was involved in the bacterial stress response and virulence, which is in accordance with the function of Fur [66, 67].

In conclusion, this study characterized HutZ from the fish pathogen *E. piscicida*. Our results showed that the expression of *hutZ*_*Ep*_ was upregulated by serum stress and was negatively regulated by Fur. HutZ_Ep_ was not involved in iron acquisition and haem utilization but played an important role in coping with adverse circumstances and functioned as a factor that was essential to bacterial infection both at the cellular level and in a live fish model. HutZ_Ep_ was also required for blocking host macrophage activation. This report is the first study of HutZ in a fish pathogen, and the results indicated that HutZ_Ep_ is a novel virulence factor of *E. piscicida*.

## Supplementary information

**Additional file 1. Multiple sequence alignment of HutZ homologues and spatial structure of HutZ.** A, Sequence alignment of *Edwardsiella piscicida* HutZ with *Escherichia coli* ChuY and its homologues from other species. The percentage number in the bracket following each species name represents the overall sequence identity between HutZ_Ep_ and the specified species. The consensus residues are in dark blue, and the residues that are ≥ 75% identical among the aligned sequences are in pink. The GenBank accession numbers of the aligned sequences are as follows: *Edwardsiella piscicida*, WP_012848635.1; *Escherichia coli*, AUG95424.1; *Citrobacter koseri*, WP_115626451.1; and *Klebsiella oxytoca*, WP_142475928.1. B, The spatial structure was determined with the PyMOL Molecular Graphics System. α-Helices are shown in red.
