# Supplementary figures and images for: HutZ is required for biofilm formation and contributes to the pathogenicity of Edwardsiella piscicida
Source: Vet Res. 2019 Oct 2;50:76. doi: 10.1186/s13567-019-0693-4 (PMC6775658; doi:10.1186/s13567-019-0693-4)

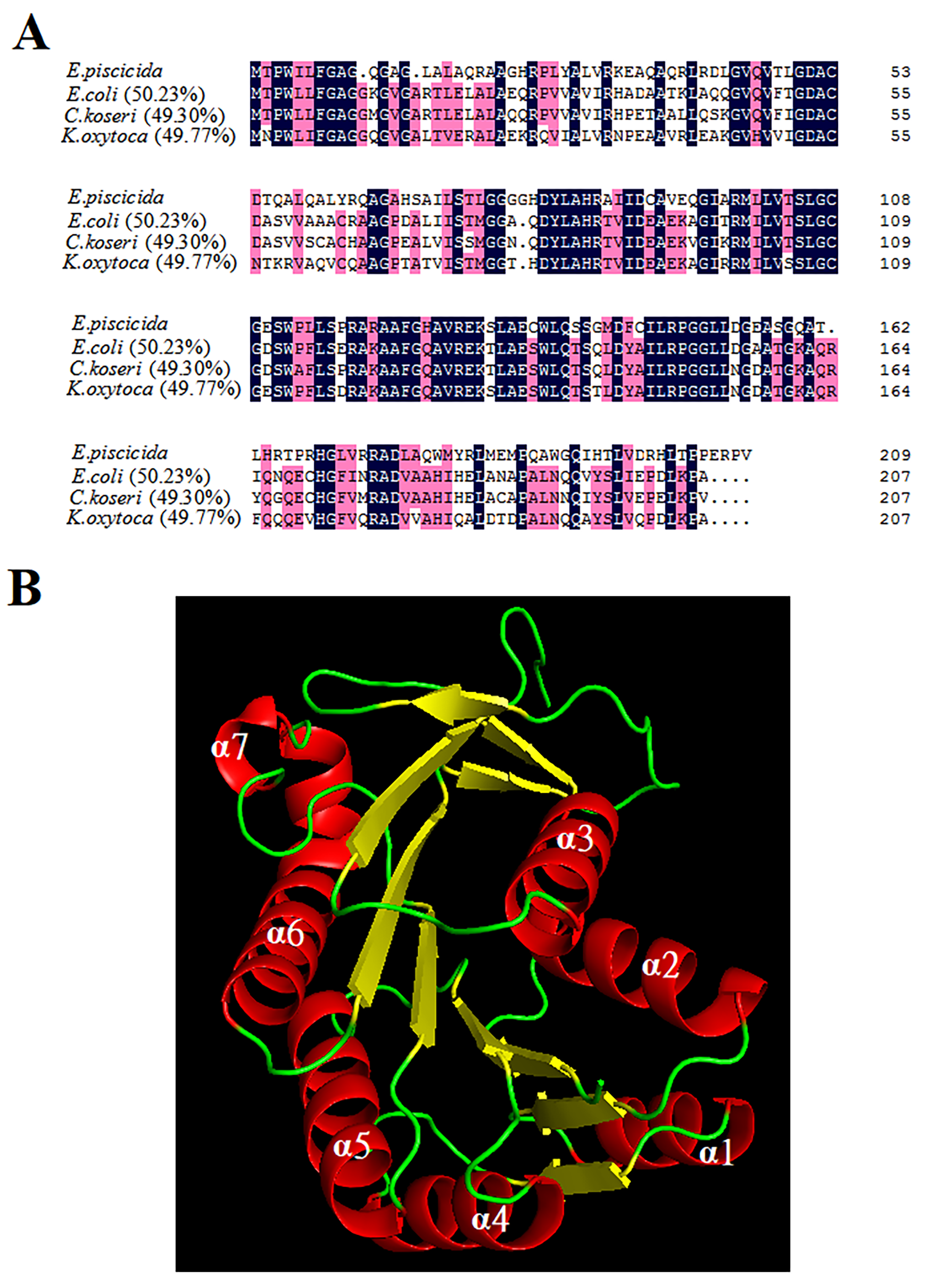

Supplement: Supplementary file 1 — Additional file 1. Multiple sequence alignment of HutZ homologues and spatial structure of HutZ. A, Sequence alignment of Edwardsiella piscicida HutZ with Escherichia coli ChuY and its homologues from other species. The percentage number in the bracket following each species name represents the overall sequence identity between HutZEp and the specified species. The consensus residues are in dark blue, and the residues that are ≥ 75% identical among the aligned sequences are in pink. The GenBank accession numbers of the aligned sequences are as follows: Edwardsiella piscicida, WP_012848635.1; Escherichia coli, AUG95424.1; Citrobacter koseri, WP_115626451.1; and Klebsiella oxytoca, WP_142475928.1. B, The spatial structure was determined with the PyMOL Molecular Graphics System. α-Helices are shown in red. [file 13567_2019_693_MOESM1_ESM.tif]
